# Microsatellite instability in thyroid tumours and tumour-like lesions

**DOI:** 10.1038/sj.bjc.6690054

**Published:** 1999-01

**Authors:** D Lazzereschi, R Palmirotta, A Ranieri, L Ottini, M C Verì, A Cama, F Cetta, F Nardi, G Colletta, R Mariani-Costantini

**Affiliations:** Department of Experimental Medicine and Pathology, University ‘La Sapienza’, Viale Regina Elena 324, Rome, 00161, ITALY; Department of Oncology and Neurosciences, University ‘G. D'Annunzio’ Chieti, Italy; Institute of Surgical Clinics, Nuovo Policlinico, V.le Bracci, Siena, 53100, Italy

**Keywords:** thyroid tumour: microsatellite instability, RER^+^ phenotype, lymph node metastasis, transforming growth factor β, type II receptor gene, insulin-like growth factor II receptor gene

## Abstract

Fifty-one thyroid tumours and tumour-like lesions were analysed for instability at ten dinucleotide microsatellite loci and at two coding mononucleotide repeats within the transforming growth factor β (TGF-β) type II receptor (*TβRII*) and insulin-like growth factor II (IGF-II) receptor (*IGFIIR*) genes respectively. Microsatellite instability (MI) was detected in 11 out of 51 cases (21.5%), including six (11.7%) with MI at one or two loci and five (9.8%) with Ml at three or more loci (RER^+^ phenotype). No mutations in the *TβRII* and *IGFIIR* repeats were observed. The overall frequency of MI did not significantly vary in relation to age, gender, benign versus malignant status and tumour size. However, widespread MI was significantly more frequent in follicular adenomas and carcinomas than in papillary and Hürthle cell tumours: three out of nine tumours of follicular type (33.3%) resulted in replication error positive (RER^+^), versus 1 out of 29 papillary carcinomas (3.4%, *P* = 0.01), and zero out of eight Hürthle cell neoplasms. Regional lymph node metastases were present in five MI-negative primary cancers and resulted in MI-positive in two cases. © 1999 Cancer Research Campaign


*British Journal of Cancer* (1999) **79**(2), 340–345
© 1999 Cancer Research Campaign

### Microsatellite instability in thyroid tumours and tumour-like lesions

**D Lazzereschi[1]*, R Palmirotta[2]*, A Ranieri[2], L Ottini[1], MC Verì[2], A Cama[2], F Cetta[3], F Nardi[1], G Colletta[2] and R Mariani-Costantini[2]**

Department of [1]Experimental Medicine and Pathology, University 'La Sapienza', Viale Regina Elena 324, 00161 Rome, and Department of [2]Oncology and Neurosciences, University 'G. D'Annunzio', Via dei Vestini, 1, 66100 Chieti, Italy; [3]Institute of Surgical Clinics, Nuovo Policlinico, University of Siena, V.le Bracci, 53100 Siena, Italy

**Summary** Fifty-one thyroid tumours and tumour-like lesions were analysed for instability at ten dinucleotide microsatellite loci and at two coding mononucleotide repeats within the transforming growth factor β (TGF-β) type II receptor (*TβRII*) and insulin-like growth factor II (IGF-II) receptor (*IGFIIR*) genes respectively. Microsatellite instability (MI) was detected in 11 out of 51 cases (21.5%), including six (11.7%) with MI at one or two loci and five (9.8%) with MI at three or more loci ($RER^+$ phenotype). No mutations in the *TβRII* and *IGFIIR* repeats were observed. The overall frequency of MI did not significantly vary in relation to age, gender, benign versus malignant status and tumour size. However, widespread MI was significantly more frequent in follicular adenomas and carcinomas than in papillary and Hürthle cell tumours: three out of nine tumours of follicular type (33.3%) resulted in replication error positive ($RER^+$), versus 1 out of 29 papillary carcinomas (3.4%, $P$ = 0.01), and zero out of eight Hürthle cell neoplasms. Regional lymph node metastases were present in five MI-negative primary cancers and resulted in MI-positive in two cases.

**Keywords:** thyroid tumour: microsatellite instability; $RER^+$ phenotype; lymph node metastasis; transforming growth factor β type II receptor gene; insulin-like growth factor II receptor gene

Thyroid tumours are the most frequent neoplasms of the endocrine system. The diverse spectrum of tumours and tumour-like lesions originating from thyroid follicular cells provides an useful model for the study of alternative pathways of epithelial tumorigenesis at the molecular level (Hedigar et al, 1988; Farid et al, 1994). Mutations activating the *RAS* proto-oncogenes represent an early step in the pathway of follicular thyroid tumorigenesis (Lemoine et al, 1989), whereas translocations involving the *RET* and *TRK* oncogenes, with consequent expression of aberrant fusion proteins as well as amplification and overexpression of the *MET* oncogene, have been implicated in papillary thyroid carcinogenesis (Bongarzone et al, 1989; Di Renzo et al, 1992). A role for tumour-suppressor genes has also been evidenced: anaplastic carcinomas have a high frequency of point mutations of the *p53* gene, and deletions of chromosome 3p22 have been found in follicular carcinomas (Fagin et al, 1993; Hermann et al, 1996). Very recently, involvement of the *PTEN*/*MMAC1* tumour-suppressor gene has been linked to the 10q22–23 loss of heterozygosity (LOH) observed in thyroid tumours of follicular histotype associated with Cowden's syndrome (Liaw et al, 1997).

Microsatellites are simple and abundantly distributed polymorphic sequence repeats that represent 'hotspots' for base-pairing errors during DNA replication (Karran, 1996). A very high incidence of tumour-associated microsatellite mutations, consisting of contractions or expansions in the number of repetitive unit sequences, was initially reported in tumours of patients with hereditary non-polyposis colorectal cancer (HNPCC) and in a subset of sporadic colorectal cancers (Peltomäki et al, 1993; Thibodeau et al, 1993). HNPCC families present also extra-colorectal malignancies, notably including endometrial, gastric and ovarian carcinomas, but not thyroid tumours (Lynch et al, 1997). In HNPCC-associated tumours, widespread microsatellite instability (MI) is correlated with double-hit inactivation of one of at least four different genes, *hMLH1*, *hMSH2*, *hPMS1* and *hPMS2*, encoding mismatch recognition and repair (MMR) proteins (Hemminki et al, 1994; Liu et al, 1994; Nicolaides et al, 1994). Various degrees of MI have also been reported in colorectal and extracolorectal cancer cell lines (Boyer et al, 1995), and in several forms of sporadic extracolorectal tumours (Eshleman and Markowitz, 1995; Karran, 1996). At present, the genetic base(s) of MI in sporadic cancers are poorly defined because MMR gene mutations have been identified only in a minority of the cases (Konishi et al, 1996).

A limited number of studies have examined the occurrence and the role of MI in thyroid tumours. The small series of nine cases reported by Vermiglio et al (1995) did not exhibit any MI, although a limited incidence of MI was observed in the larger series of tumours and tumour-like lesions analysed by Soares et al (1997). To explore the role of MI in thyroid carcinogenesis, we analysed the status of ten dinucleotide repeat microsatellite loci in a panel of 51 tumours and tumour-like lesions. We also tested the occurrence of mutations at two coding mononucleotide repeats within the transforming growth factor β (TGF-β) type II (*TβRII*) and insulin-like growth factor II (IGF-II) (*IGFIIR*) receptor genes

*Both authors equally contributed to this work.

*Received 14 October 1997*
*Revised 21 April 1998*
*Accepted 6 May 1998*

*Correspondence to:* R Mariani-Costantini, Laboratory of General Pathology, Department of Oncology and Neurosciences, University 'Gabriele D'Annunzio', Via dei Vestini 1, 66013 Chieti, Italy

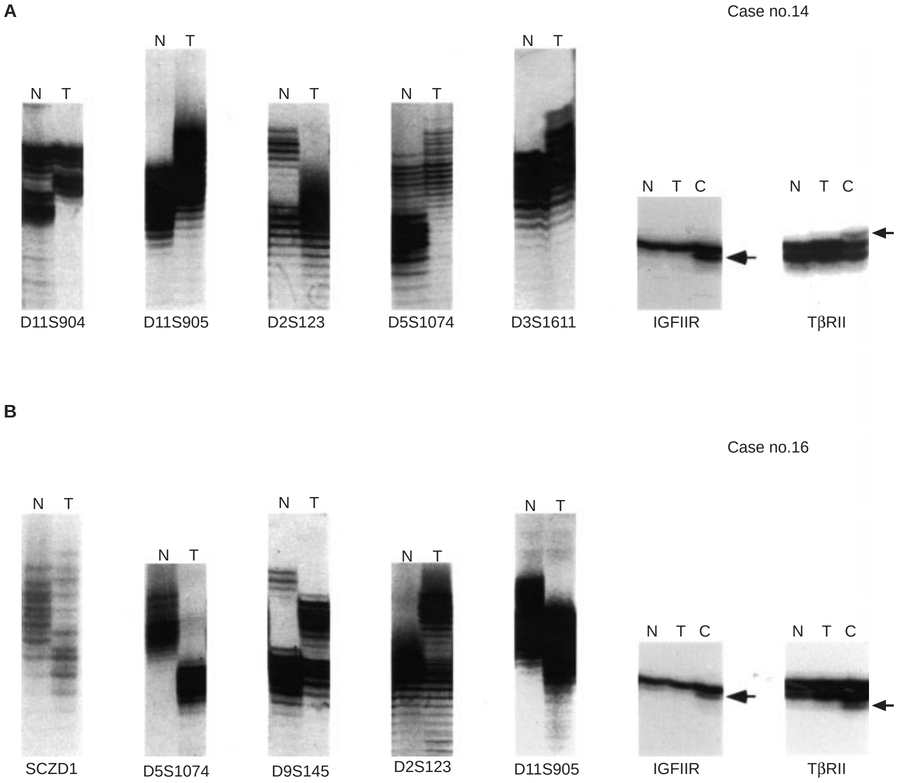

**Figure 1** Microsatellite alterations and status of the *TβRII* poly-$A_{10}$ and *IGFIIR* poly-$G_8$ mononucleotide repeats in paired normal thyroid (N) and tumour (T) DNAs from papillary carcinoma case no. 14 (**A**) and anaplastic carcinoma case no. 16 (**B**). **A** and **B**, corresponding to each microsatellite marker, depict a paired genotyping that presented novel tumour-associated alleles originating from compressions or expansions of repeats. Compressions of repeats are exemplified at D2S123 in case no. 14 and, in case no. 16, at SCZD1 and D9S145, in which instability involves both alleles, and at D11S905 and D5S1074, in which instability is associated to loss of one allele or co-migration of both alleles. Expansions of repeats are exemplified in case no. 14 at D11S904, D11S905, D5S1074 and D3S1611, and, in case no. 16, at D2S123. The banding pattern of the *TβRII* poly-$A_{10}$ and *IGFIIR* poly-$G_8$ sequences is normal in both thyroid tumours (T), as compared with the respective normal thyroid controls (N) and to a mutated gastric cancer control (C)

respectively. Mutations at these repeats result in the inactivation of the *TβRII* and/or *IGFIIR* gene products and represent frequent events in MI-positive gastrointestinal malignancies (Markowitz et al, 1995; Myeroff et al, 1995; Parson et al, 1995; Souza et al, 1996; Ottini et al, 1998). In the present study, widespread MI tended to be associated with lesions of the follicular type (LiVolsi, 1990). There were no mutations at the mononucleotide repeats within the *TβRII* and *IGFIIR* genes. The analysis of lymph node metastases revealed that MI may occur during thyroid tumour progression.

### MATERIALS AND METHODS

### Pathological samples and clinical data

Forty-eight cases of sporadic thyroid neoplasms from unrelated patients were diagnosed, classified and staged according to the UICC criteria (Hermanek and Sobin, 1987) by one of the authors (FN). Forty-one fresh paired normal thyroid and tumour samples (cases no. 1–41) were obtained from partial or total thyroidectomy specimens surgically removed during the last 3 years at the University ‘La Sapienza’, Rome, Italy. To minimize the possibility of an inadvertent inclusion of normal tissue in the tumour samples and vice versa, samples were selected from microscopically controlled areas. The samples were promptly frozen and stored at –80°C until DNA extraction. Seven additional cases of sporadic thyroid tumours were retrieved from surgical pathology files (cases AR1–AR7). Moreover, three papillary thyroid carcinomas occurring in distinct familial adenomatous polyposis (FAP) patients from a single FAP kindred (Civitelli et al, 1996) were also analysed. Altogether, the 51 cases analysed comprised three follicular nodules, considered as putative follicular preneoplastic lesions (LiVolsi, 1990), seven follicular adenomas, including two microfollicular and one trabecular variants, six Hürthle cell adenomas, two follicular carcinomas, two Hürthle cell carcinomas, two anaplastic carcinomas and 29 papillary carcinomas, two of which contained areas with follicular differentiation. Regional lymph node metastases were analysed in five cases, including a Hürthle cell carcinoma (AR7) with 14 distinct metastases and four papillary carcinomas: case AR2, with three metastases; cases AR5 and no. 11, with two metastases; and case no. 29, with a single metastasis.

### DNA extraction

Extraction of normal tissue and tumour DNA from fresh samples was performed using standard methods (Lazzereschi et al, 1997). For formalin-fixed, paraffin-embedded samples, sections, 5–10 μm in thickness, were collected on glass slides and stained with haematoxylin. After pathological review, areas of normal tissue and tumour were marked and microdissected; where the areas of interest could not be clearly separated from surrounding tissue, selective ultraviolet radiation fractionation (Shibata et al, 1996)

**Table 1** Clinicopathological characteristics of the primary and metastatic thyroid tumours and tumour-like lesions with evidence of microsatellite instability (MI)

| Code no. | Gender/ age | Type | TNM | Clinical stage | Size (cm) | Origin of sample[a] | MI |
|---|---|---|---|---|---|---|---|
| 37 | M/49[b] | Follicular nodule | NA[c] | NA | 1.0 | T | D2S123 |
| 2 | M/24 | Follicular adenoma | NA | NA | 3.5 | T | 635/636, D2S174, D5S1074, D11S904 |
| 9 | W/37[b] | Follicular adenoma | NA | NA | 3.0 | T | 635/636, D9S145, SCZD1 |
| AR6 | W/35 | Follicular carcinoma | T4NxMx | I | 3.5 | T | D2S123, D2S174, D3S1611, D5S1074, D9S145, SCZD1, D11S905 |
| AR7 | M/74 | Hürthle cell carcinoma | T1N2Mx | II | ND[d] | T | – |
| | | | | | < 1 | M1 | D11S905 |
| | | | | | 2 | M2 | D3S1611 |
| | | | | | 1.8 | M3 | D9S145 |
| | | | | | 0.7 | M4 | D9S145 |
| | | | | | 1.2 | M5 | D2S174, D17S250 |
| 1 | W/26 | Papillary carcinoma | T4N0Mx | III | 3.0 | T | 635/636 |
| 14 | M/32 | Papillary carcinoma | T1N0Mx | I | < 1 | T | D2S123, D3S1611, D5S1074, D11S904, D11S905 |
| 25 | W/32 | Papillary carcinoma | T1NxMx | I | 0.8 | T | 635/636 |
| 27 | M/70 | Papillary carcinoma | T2NxMx | II | 1.7 | T | D11S904 |
| AR1 | W/ND | Papillary carcinoma | T2N1Mx | ND | 1.2 | T | D9S145, D11S905 |
| AR4 | W/32 | Papillary carcinoma | T2NxMx | I | 1.2 | T | D11S904 |
| AR5 | M/40 | Papillary carcinoma | T2N1Mx | I | ND | T | – |
| | | | | | 1.2 | M1 | D9S145 |
| 16 | W/72 | Anaplastic carcinoma | T4NxMx | IV | ND | T | D2S123, D5S1074, D9S145, SCZD1, D11S905 |

[a]T, primary thyroid tumour or tumour-like lesion; M1–5, different lymph node metastases. [b]M, man; W, woman. [c]NA, not applicable. [d]ND, not determined.

was performed. Genomic DNA from microdissected paraffin-embedded samples was extracted as previously described (Ottini et al, 1995, 1997). One microlitre (1 µl) of DNA was used to set up 10-ml polymerase chain reactions (PCR).

### Microsatellite analysis

For microsatellite typings, a two-step protocol was used consisting of a non-radioactive external PCR, followed by a radioactive internal PCR (nested PCR) and utilizing a 1:$10^4$ dilution of primary PCR products as template. The following ten microsatellite dinucleotide repeats were analysed: *D2S123*, *D2S174*, *D3S1611*, *SCZD1*, *D5S1074*, *D9S145*, *D11S904*, *D11S905*, *635–636* and *D17S250*. Primers, PCR mixture, cycling conditions, electrophoretic separation and autoradiography were as previously described (Ottini et al, 1995). The 73-bp region encompassing the poly-$A_{10}$ repeat of the *TβRII* gene at nucleotides 709–718 and the 110-bp region including the poly-$G_8$ tract of the *IGFIIR* gene at nucleotides 4089–4096 were amplified by using previously reported primer sets (Parson et al, 1995; Souza et al, 1996). The presence of mutations was tested using mutated MI-positive gastric cancers as positive controls (Ottini et al, 1998). PCR mixture, cycling conditions, electrophoretic separation and autoradiography were as previously used for MI analysis (Ottini et al, 1995).

Statistical analyses for the correlation of microsatellite alterations with clinicopathological characteristics were performed using the $\chi^2$ test. The *P*-values were computed after combining the fraction of cases with MI at three or more loci, versus the fraction of cases with no evidence of MI and with MI limited to one or two loci. Statistical significance was considered at $P \leq 0.05$.

### RESULTS

A series of thyroid tumours and tumour-like lesions, paired to their associated normal tissues, was tested for MI at ten dinucleotide repeat microsatellite loci. The same samples were also analysed for the presence of coding repeat mutations at a poly-$A_{10}$ tract within the *TβRII* gene (nucleotides 709–718) and at a poly-$G_8$ tract within the *IGFIIR* gene (nucleotides 4030–4140). All MI-positive typings were confirmed in duplicate or triplicate experiments, using samples from independent DNA extractions. Allelic imbalances were not considered in the present study.

Out of the 51 primary thyroid lesions analysed, 11 (21.5%) were positive for MI, as identified by the presence of tumour-associated electrophoretic mobility shifts of microsatellite alleles at one or more loci. MI-positive samples included one out of three follicular nodules, two out of seven follicular adenomas, 6 out of 29 papillary carcinomas, one out of two follicular carcinomas and one out of two anaplastic carcinomas. Interestingly, the three FAP-associated papillary carcinomas were MI negative.

No abnormally migrating bands indicative of mutations in the *TβRII* poly-$A_{10}$ and *IGFIIR* poly-$G_8$ sequences were detected in the 51 cases examined, including the five tumours with MI at three or more loci. In spite of the absence of mutations at the *TβRII* poly-$A_{10}$ repeat, it is noteworthy that 7 out of 11 MI-positive cases reported in this study, including three out of five cases with MI at three or more loci, showed reduced *TβRII* expression at the RNA and/or protein level, as demonstrated in a previous work (Lazzereschi et al, 1997). Figure 1 shows paired normal tissue and tumour microsatellite genotypings from two cases, papillary carcinoma no. 14 and anaplastic carcinoma no. 16, that manifested MI at five out of ten loci but had a normal banding pattern of the *TβRII* poly-$A_{10}$ and *IGFIIR* poly-$G_8$ sequences compared with mutated gastric cancer controls. Notably, anaplastic carcinoma no. 16 previously demonstrated reduced *TβRII* expression (Lazzereschi et al, 1997).

The MI status of regional lymph node metastases could be examined after microdissection of metastatic foci and compared with that of the corresponding primary carcinoma in five cases. As

 © Cancer Research Campaign 1999

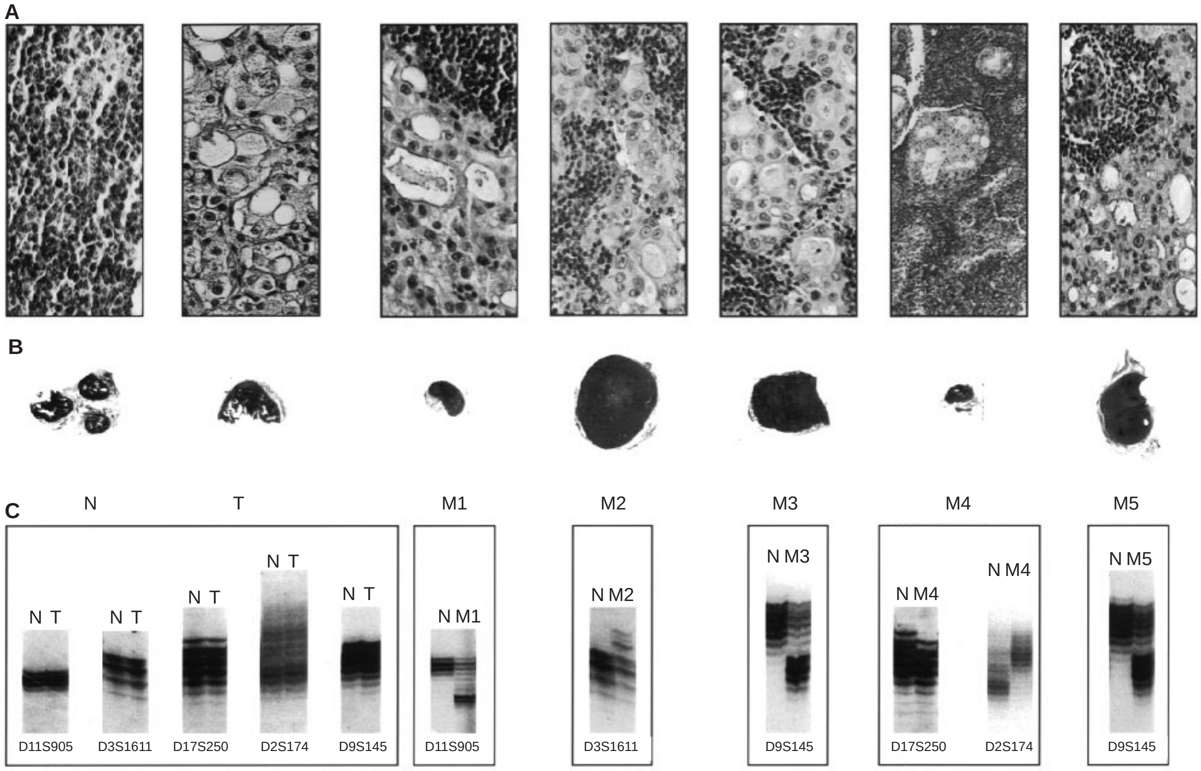

**Figure 2** Microsatellite profiles of primary tumour (T), matched normal tissue (N) and individual regional lymph node metastases (M1, M2, M3, M4, M5) from Hürthle cell carcinoma AR7. **A** and **B** respectively show the histology and low magnification views of the samples that were individually analysed after microdissection, **C** shows the banding patterns of the microsatellite markers that demonstrated MI in lymph node metastases. The primary tumour does not show any microsatellite alteration, as compared with normal thyroid tissue. The five metastases depicted here exhibit heterogeneous MI patterns at D11S905 (M1), D3S1611 (M2), D9S145 (M3 and M5, both sharing the same pattern), D2S714 and D17S250 (M4)

reported in Table 1, metastases from two MI-negative primary cancers, a papillary and a Hürthle cell carcinoma, were MI positive. In particular, the unique lymph node metastasis of papillary carcinoma AR5 was MI positive at D9S145, whereas 5 out of 14 metastases from Hürthle cell carcinoma AR7 demonstrated heterogeneous MI patterns, indicative of clonal metastatic diversity. Figure 2 shows relevant microsatellite profiles from case AR7: metastases designated M1, M2, M3 and M5 demonstrated MI at one locus, corresponding to D11S905 (M1), D3S1611 (M2) and D9S145 (M3 and M5 both sharing the same MI pattern), metastasis M4 was MI positive at loci D2S174 and D17S250.

It is presently accepted that only widespread MI, defined by the presence of instability at 30% or more of the loci analysed and referred to as replication error-positive ($RER^+$) phenotype, is associated with a distinct clinicopathological profile (Eshleman and Markowitz, 1996; Karran, 1996). In the present study, 5 out of 51 (9.8%) cases of primary thyroid tumours and tumour-like lesions, comprising two follicular adenomas, one follicular carcinoma, one anaplastic carcinoma and one papillary carcinoma, manifested MI at three or more loci. The remaining six MI-positive cases (11.7%), including one solitary follicular nodule and five papillary carcinomas, manifested MI limited to one or two loci (Table 1). No preference for instability at any specific microsatellite locus was observed: in fact, seven out of the ten loci analysed were altered at the same frequency. Table 2 reports the fractions of primary tumours and tumour-like lesions with low (one or two loci) and high (three or more loci) MI according to a series of clinicopathological features. MI was not exclusive to malignancies because it was detected in a solitary follicular nodule and in two follicular adenomas. In particular, MI at three or more loci was observed in 2 out of 16 benign lesions (12.5%) and in 3 out of 35 carcinomas (8.6%). High-level MI (three or more loci) tended to be associated with lesions of follicular type: in fact, three out of nine (33.3%) tumours with follicular growth pattern, including two out of seven follicular adenomas and one out of two follicular carcinomas, manifested MI at three or more loci, versus 1 out of 29 (3.4%) papillary carcinomas ($P = 0.01$) and zero out of eight Hürthle cell tumours which included six adenomas and two carcinomas (Tables 1 and 2). Moreover, MI at three or more loci was detected in one out of two anaplastic carcinomas, usually considered poorly differentiated lesions of follicular lineage (Werner and Ingbar, 1991). With regard to clinical stage, 5 out of 29 carcinomas of stages I or II manifested MI, including three cases with MI at one or two loci and two cases with MI at three or more loci, compared with two out of five tumours of stages III or IV, including a papillary carcinoma with MI at one locus and an anaplastic carcinoma with MI at three or more loci. Differences in MI positivity in relation to patients' gender and age or tumour size were unremarkable.

### DISCUSSION

In spite of their common origin from follicular epithelium, the great variety of histotypes and the differences in biological behaviour of thyroid tumours and tumour-like lesions suggest

© Cancer Research Campaign 1999

**Table 2** Fractions of cases with microsatellite instability (MI) at one or two and three or more loci according to selected clinicopathological features in the 51 primary thyroid tumours and tumour-like lesions examined

| | | Cases with MI | |
|---|---|---|---|
| | **Men** | **1 or 2 loci** | **≥ 3 loci** |
| Gender | Men | 2/16 | 2/16 |
| | Women | 4/35 | 3/35 |
| Age (years) | < 45 | 3/33 | 4/33 |
| | ≥ 45 | 2/13 | 1/13 |
| | Unknown | 1/5 | 0/5 |
| Type of lesion | Benign | 1/16 | 2/16 |
| | Malignant | 5/35 | 3/35 |
| Tumour size | ≤ 1 cm | 2/12 | 1/12 |
| | ≤ 4 cm | 4/32 | 3/32 |
| | > 4 cm | 0/4 | 0/4 |
| | Unknown | 0/3 | 1/3 |
| Histological pattern | Nodule[a] | 1/3 | 0/3 |
| | Follicular[b] | 0/9 | 3/9 *P* 0.01 |
| | Hürthle cell[b] | 0/8 | 0/8 |
| | Papillary | 5/29 | 1/29 |
| | Anaplastic | 0/2 | 1/2 |
| Clinical stage | I | 2/25 | 2/25 |
| | II | 1/4 | 0/4 |
| | III | 1/2 | 0/2 |
| | IV | 0/3 | 1/3 |
| | Unknown | 1/1 | 0/1 |

[a]Follicular nodule. [b]Adenomas and carcinomas combined.

different pathways of thyroid tumorigenesis (Hedigar et al, 1988; Werner and Ingbar, 1991). This is consistent with the existence of alternative genetic pathways for tumour development and progression, confirmed at the molecular level by distinct gene alterations in the papillary and in the follicular histotypes (Farid et al, 1994; Wynford-Thomas, 1997).

Microsatellite instability is an indicator of decreased replication fidelity of genomic DNA, which, when at multiple loci, is believed to be associated with genetic defects that promote tumorigenesis (Eshleman and Markowitz, 1996; Karran, 1996). Few works have analysed the MI status of thyroid tumours and tumour-like lesions. A preliminary analysis based on a small series of cases, tested at a limited number of microsatellite markers, failed to evidence any MI (Vermiglio et al, 1995), whereas a second report was suggestive of a limited role of MI in both benign and malignant tumours and even in non-neoplastic lesions such as goitres (Soares et al, 1997). To further investigate the frequency and role of MI in thyroid carcinogenesis, a panel of cases encompassing all the most common tumours and tumour-like lesions has been examined for MI at ten dinucleotide repeats. Overall, MI at one or more loci was found in 21.5% of thyroid tumours, a frequency comparable to those reported for tumours of other origins (Thibodeau et al, 1993; Eshleman and Markowitz, 1995; Karran, 1996). Widespread MI, i.e. at 30% or more of the loci (RER$^+$ phenotype), was found in 9.8% of the thyroid tumours, corresponding to 45% of all MI-positive cases.

It is noteworthy that cases with MI at multiple loci were significantly more frequent in follicular type tumours and tumour-like lesions relative to papillary and Hürthle cell-type tumours. Interestingly, three FAP-associated thyroid papillary carcinomas, occurring in carriers of a germline mutation at codon 1061 of the adenomatous polyposis coli (APC) gene (Civitelli et al, 1996), resulted MI negative. The occurrence of widespread MI in benign thyroid lesions and its low frequency in papillary carcinomas were in agreement with the results of Soares et al (1997). There were no associations between MI status and patients' age and gender or tumour size.

The comparison of the microsatellite profiles of lymph node metastases with those of primary thyroid tumours and matched normal tissues revealed that metastases with heterogeneous MI-positive patterns may originate from primary cancers with no evidence of MI. As in gastrointestinal cancer and melanoma (Shibata et al, 1996; Richetta et al, 1997), this indicates either dominant metastatic expansion of tumour cell clones underrepresented in the primary lesion, or diversification of microsatellite alleles and clonal evolution during population doublings after metastasization. The occurrence of MI in metastases from MI-negative primary tumours and the presence of widespread MI in two out of five high-stage primary carcinomas raises the possibility that genomic instability at sequence repeats might be associated with tumour progression in some thyroid cancers. Further investigations of larger series of cases are required to verify this hypothesis.

TGF-β is the most important growth-inhibitory factor in thyroid cells (Colletta et al, 1989), and resistance or escape from TGF-β control could contribute to the neoplastic transformation of follicular epithelium (Blaydes and Wynford-Thomas, 1996; Brattain et al, 1996; Coppa et al, 1997). *TβRII* mutations could have been responsible for the loss of TGF-β responsiveness and for the reduced levels of *TβRII* mRNA and protein that we previously found in thyroid malignancies, including cases investigated in this study (Lazzereschi et al, 1997). Nevertheless, none of the presently analysed 51 primary tumours and tumour-like lesions, including the 11 MI-positive cases, revealed mutations in the *TβRII* and *IGFIIR* coding repeats analysed, which represent frequent sites of inactivating mutations in MI-positive gastrointestinal cancer (Markowitz et al, 1995; Myeroff et al, 1995; Parson et al, 1995; Souza et al, 1996; Ottini et al, 1998). This suggests that in thyroid tumorigenesis loss or down-regulation of *TβRII* expression occurs via MI-independent mechanisms.

In conclusion, our results suggest that MI may influence the biology of follicular thyroid tumours and tumour-like lesions and the molecular mechanisms of progression of thyroid cancer in general. This warrants further research on the genetic base(s) and functional consequences of genomic instability in thyroid carcinogenesis.

### ACKNOWLEDGEMENTS

Study supported by the CNR-ACRO grant no. 94.01161.PF39 and by AIRC grants. LO is an AIRC fellow.

### REFERENCES

Blaydes JP and Wynford-Thomas D (1996) Loss of responsiveness to transforming growth factor β is tightly linked to tumorigenicity in a model of thyroid tumour progression. *Int J Cancer* **65**: 525–530

Bongarzone I, Pierotti MA, Monzini N, Mondellini P, Manenti G, Donghi R, Pilotti S, Grieco M, Santoro M, Fusco A, Vecchio G and Della Porta G (1989) High frequency of activation of tyrosine kinase oncogenes in human papillary thyroid carcinoma. *Oncogene* **4**: 1457–1462

Boyer JC, Umar A, Risinger JI, Lipford JR, Kane M, Yin S, Barrett JC, Kolodner RD and Kunkel TA (1995) Microsatellite instability, mismatch repair deficiency and genetic defects in human cancer cell lines. *Cancer Res* **55**: 6063–6070

 © Cancer Research Campaign 1999

Brattain MG, Markowitz SD and Willson JKV (1996) The type II transforming growth factor β receptor as a tumour suppressor gene. *Curr Opin Oncol* **8**: 49–53

Civitelli S, Taurini G, Cetta F, Petracci M, Pacchiarotti MC and Civitelli B (1996) Papillary thyroid carcinoma in 3 siblings with familial adenomatous polyposis. *Int J Colorectal Dis* **11**: 571–574

Colletta G, Cirafici AM and Di Carlo A (1989) Dual effect of TGFβ on rat thyroid cells: inhibition of thyrotropin induced proliferation and reduction of thyroid-specific differentiation markers. *Cancer Res* **49**: 3457–3462

Coppa A, Mincione G, Lazzereschi D, Ranieri A, Turco A, Lucignano B, Scarpa S, Ragano-Caracciolo M and Colletta G (1997) Restored expression of Transforming Growth Factor β type II receptor in k-*ras*-transformed thyroid cells, TGFβ-resistant, reverts their malignant phenotype. *J Cell Physiol* **172**: 200–208

Di Renzo MF, Olivero M, Ferro S, Prat M, Bongarzone I, Pilotti S, Belfiore A, Costantino A, Vigneri R and Pierotti MA (1992) Overexpression of the c-MET/HGF receptor gene in human thyroid carcinomas. *Oncogene* **7**: 2549–2553

Eshleman JR and Markowitz SD (1995) Microsatellite instability in inherited and sporadic neoplasms. *Curr Opin Oncol* **7**: 83–89

Eshleman JR and Markowitz SD (1996) Mismatch repair defects in human carcinogenesis. *Hum Mol Genet* **5**: 1486–1494

Fagin JA, Matsuo K, Karkamar A, Chen DL, Tang S-H and Koeffler HP (1993) High frequency of mutations of the p53 gene in poorly differentiated human thyroid carcinomas. *J Clin Invest* **91**: 179–184

Farid NR, Shy Y and Zou M (1994) The molecular biology of thyroid cancer. *Endocr Rev* **15**: 202–232

Hedigar C, Williams ED and Sobin LH (1988) *Histological Typing of Thyroid Tumours*. Springer-Verlag: Berlin

Hemminki A, Peltomäki P, Leach FS, Sistonen P, Pylkkanen L, Meklin J-P, Järvinen H, Salovaara R, Nyström-Lahti M, De La Chapelle A and Aaltonen LA (1994) Loss of wild type hMLH1 gene is a feature of hereditary nonpolyposis colorectal cancer. *Nature Genet* **8**: 405–410

Hermanek P and Sobin LH (1987) *TNM Classification of Malignant Tumours*. UICC: Geneva

Hermann MA, Hay ID, Bartelt DH, Ritland SR, Dahl R, Grant CS and Jenkins RB (1996) Cytogenetic and molecular genetic studies of follicular and papillary thyroid cancers. *J Clin Invest* **88**: 1596–1604

Karran P (1996) Microsatellite instability and DNA mismatch repair in human cancer. *Semin Cancer Biol* **7**: 15–24

Konishi M, Kikuchi-Yanoshita R, Tanaka K, Muraoka M, Onda A, Okumura Y, Kishi N, Iwama T, Mori T, Koike M, Uishio K, Chiba M, Nomizu S, Konishi F, Utsunomiya S and Miyaki M (1996) Molecular nature of colon tumours in hereditary nonpolyposis colon cancer, familial polyposis, and sporadic cancer. *Gastroenterology* **111**: 307–317

Lazzereschi D, Ranieri A, Mincione G, Taccogna S, Nardi F and Colletta G (1997) Human malignant thyroid tumours displayed reduced levels of TGFβ receptor type II mRNA and protein. *Cancer Res* **57**: 2071–2076

Lemoine NR, Mayall ES, Wyllie FS, Williams ED, Goins M, Stringer B and Wynford-Thomas D (1989) High frequency of ras oncogene activation in all stages of human thyroid tumorigenesis. *Oncogene* **4**: 159–164

Liaw D, Marsh DJ, Li J, Dahia PLM, Wang SI, Zheng Z, Bose S, Call KM, Tsou HC, Peacocke M, Eng C and Parsons R (1997) Germline mutations of the *PTEN* gene in Cowden disease, an inherited breast and thyroid cancer syndrome. *Nature Genet* **16**: 64–67

LiVolsi V (1990) *Surgical Pathology of the Thyroid*, pp. 173–212. Saunders: Philadelphia

Liu B, Parson RE, Hamilton SR, Petersen GM, Lynch HT, Watson P, Markowitz S, Wilson JKV, Green J, De La Chapelle A, Kinzler KW and Vogelstein B (1994) hMSH2 mutations in hereditary nonpolyposis colorectal cancer kindreds. *Cancer Res* **54**: 4590–4594

Lynch HT, Smyrk T and Lynch J (1997) An update of HNPCC (Lynch Syndrome). *Cancer Genet Cytogenet* **93**: 84–99

Markowitz S, Wing J, Myeroff L, Parsons R, Sun LZ, Lutterbaugh J, Fan RS, Zborowska E, Kinzler KW, Vogelstein B, Brattain M and Willson JKV (1995) Inactivation of the type II TGF-β receptor in colon cancer cells with microsatellite instability. *Science* **268**: 1336–1338

Myeroff LL, Parsons R, Kim SJ, Hedrick L, Cho KR, Orth K, Mathis M, Kinzler KW, Lutterbaugh J, Park K, Bang Y-J, Lee HY, Park J-G, Lynch H, Roberts AB, Vogelstein B and Markowitz SD (1995) A transforming growth factor β receptor type II gene mutation common in colon and gastric but rare in endometrial cancers with microsatellite instability. *Cancer Res* **55**: 5545–5547

Nicolaides NC, Papadopoulos N, Liu B, Wei YF, Carter KC, Ruben SM, Rosen CA, Haseltine WA, Fleischmann RD, Fraser CM, Adams MD, Venter CJ, Dunlop MG, Hamilton SR, Petersen GM, De La Chapelle A, Vogelstein B and Kinzler KW (1994) Mutations of two PMS homologues in hereditary nonpolyposis colon cancer. *Nature* **371**: 75–80

Ottini L, Esposito DL, Richetta A, Carlesimo M, Palmirotta R, Verì MC, Battista P, Frati L, Caramia FG, Calvieri S, Cama A and Mariani-Costantini R (1995) Alterations of microsatellites in neurofibromas of von Recklinghausen's disease. *Cancer Res* **55**: 5677–5680

Ottini L, Palli D, Falchetti M, D'Amico C, Amorosi A, Saieva C, Calzolari A, Cimoli F, Tatarelli C, De Marchis L, Masala G, Mariani-Costantini R and Cama A (1997) Microsatellite instability in gastric cancer is associated with tumour location and family history in a high risk population from Tuscany. *Cancer Res* **57**: 4523–4529

Ottini L, Falchetti M, D'Amico C, Amorosi A, Saieva C, Masala G, Frati L, Cama A, Palli D and Mariani-Costantini R (1998) Mutations at coding mononucleotide repeats in gastric cancer with the microsatellite mutator phenotype. *Oncogene* **16**: 2767–2772

Parson R, Myeroff LL, Liu B, Willson JKV, Markowitz SD, Kinzler KW and Vogelstein B (1995) Microsatellite instability and mutation of the transforming growth factor β type II receptor gene in cancers. *Cancer Res* **55**: 5548–5550

Peltomäki P, Lothe RA, Aaltonen LA, Pylkkänen L, Nystrom-Lahti MN, Seruca R, David L, Holm R, Ryberg D, Haugen A, Brøgger A, Børresen A-L and De La Chapelle A (1993) Microsatellite instability is associated with tumours that characterize the hereditary non-polyposis colorectal carcinoma syndrome. *Cancer Res* **53**: 5853–5855

Richetta A, Ottini L, Frati L, Cama A, Mariani-Costantini R and Calvieri S (1997) Microsatellite instability in primary and metastatic melanoma. *J Invest Dermatol* **109**: 119–120

Shibata D, Navidi W, Salovaara R, Li Z-H and Aaltonen LL (1996) Somatic microsatellite mutations as molecular tumour clocks. *Nature Med* **2**: 676–681

Soares P, dos Santos NR, Seruca R, Lothe RA and Sobrinho-Simões M (1997) Benign and malignant thyroid lesions show instability at microsatellite loci. *Eur J Cancer* **33**: 293–296

Souza RF, Appel R, Yin J, Wang S, Smolinski KN, Abraham JM, Zou TT, Shi YQ, Lei J, Cottrell J, Cymes K, Biden K, Simms L, Legget B, Lynch PM, Frazier M, Powell SM, Harpaz N, Sugimura H, Young J and Meltzer SJ (1996) Microsatellite instability in the insulin-like growth factor II receptor gene in gastrointestinal tumours. *Nature Genet* **14**: 255–257

Thibodeau SN, Bren G and Schaid D (1993) Microsatellite instability in cancer of the proximal colon. *Science* **260**: 816–819

Vermiglio F, Schlumberger M, Lazar V, Lefreré I and Bressac B (1995) Absence of microsatellite instability in thyroid carcinomas. *Eur J Cancer* **31A**: 128

Werner S and Ingbar SH (1991) *The Thyroid-A Fundamental and Clinical Text*, 6th edn. Braverman LE and Utiger RD (eds), pp. 1121–1206. JB Lippincott: Philadelphia

Wynford-Thomas D (1997) Origin and progression of thyroid epithelial tumours: cellular and molecular mechanisms. *Horm Res* **47**: 145–157

© Cancer Research Campaign 1999
